# Supplementary material for: Development of Approaches for Transgene Expression in the Pathogenic Free-Living Amoeba Naegleria fowleri
Source: Pathogens. 2025 Dec 22;15(1):12. doi: 10.3390/pathogens15010012 (PMC12845041; doi:10.3390/pathogens15010012)
Supplement: Supplementary file 1 [file pathogens-15-00012-s001.zip › Supplmental Figure S1_.pptx]

## Slide 1
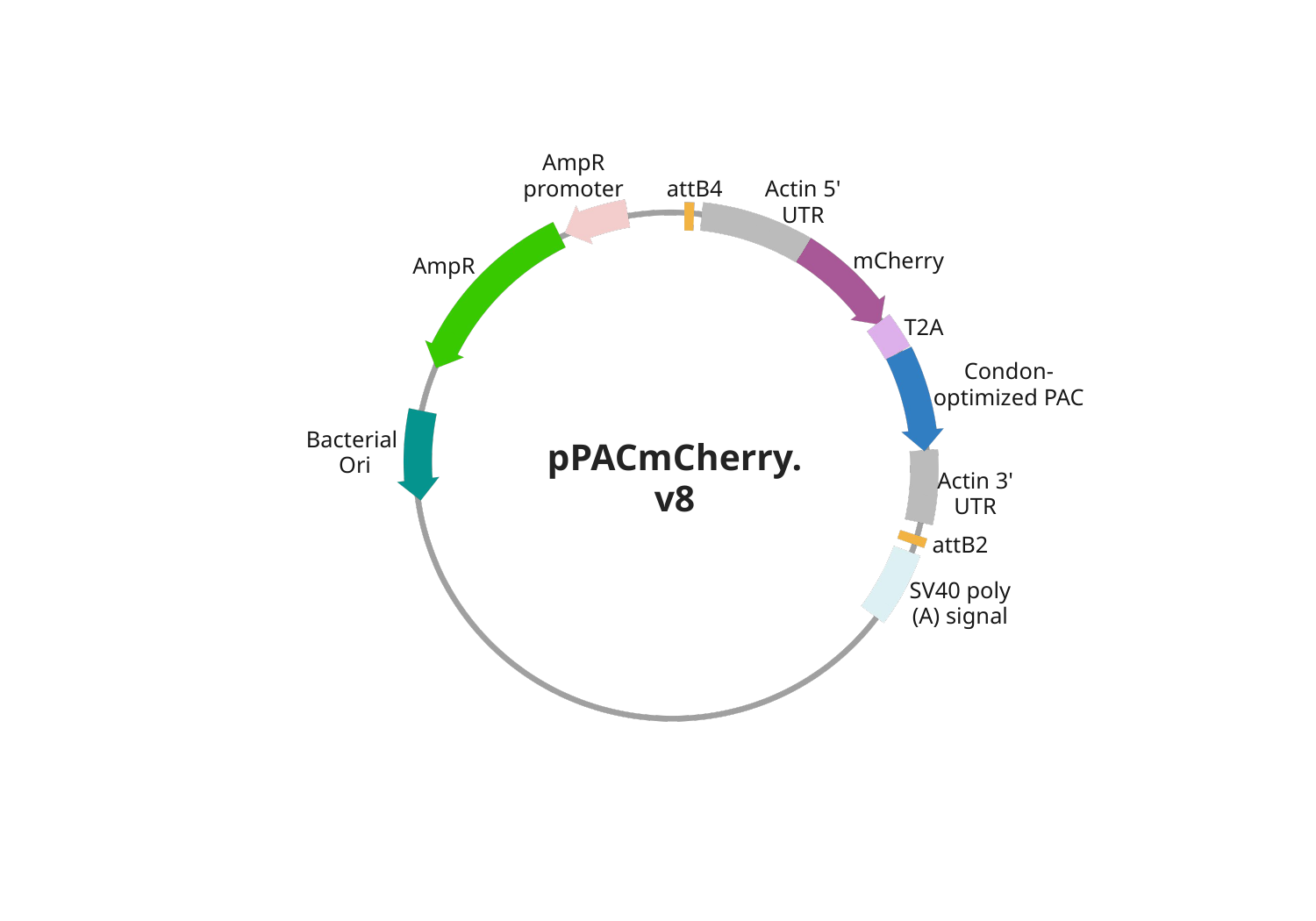

AmpR promoter
attB4
Actin 5' UTR
mCherry
AmpR
T2A
Condon-optimized PAC
Bacterial
Ori
pPACmCherry.v8
Actin 3' UTR
attB2
SV40 poly (A) signal

## Slide 2
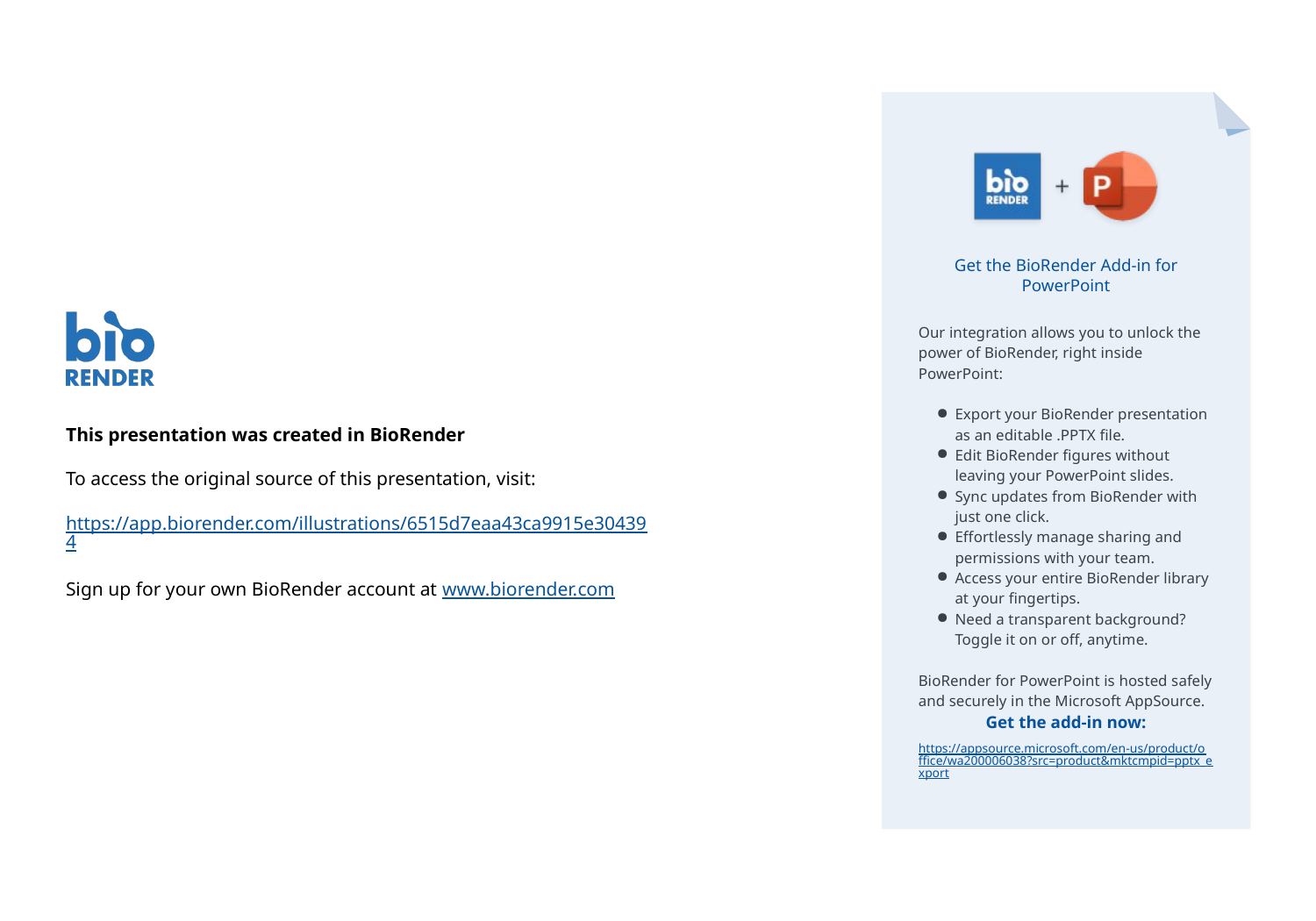

Get the BioRender Add-in for PowerPoint
Our integration allows you to unlock the power of BioRender, right inside PowerPoint:
Export your BioRender presentation as an editable .PPTX file.
Edit BioRender figures without leaving your PowerPoint slides.
Sync updates from BioRender with just one click.
Effortlessly manage sharing and permissions with your team.
Access your entire BioRender library at your fingertips.
Need a transparent background? Toggle it on or off, anytime.
BioRender for PowerPoint is hosted safely and securely in the Microsoft AppSource.
This presentation was created in BioRender
To access the original source of this presentation, visit:
https://app.biorender.com/illustrations/6515d7eaa43ca9915e304394
Sign up for your own BioRender account at www.biorender.com
Get the add-in now:
https://appsource.microsoft.com/en-us/product/office/wa200006038?src=product&mktcmpid=pptx_export
