# Supplementary figures and images for: Development of Approaches for Transgene Expression in the Pathogenic Free-Living Amoeba Naegleria fowleri
Source: Pathogens. 2025 Dec 22;15(1):12. doi: 10.3390/pathogens15010012 (PMC12845041; doi:10.3390/pathogens15010012)

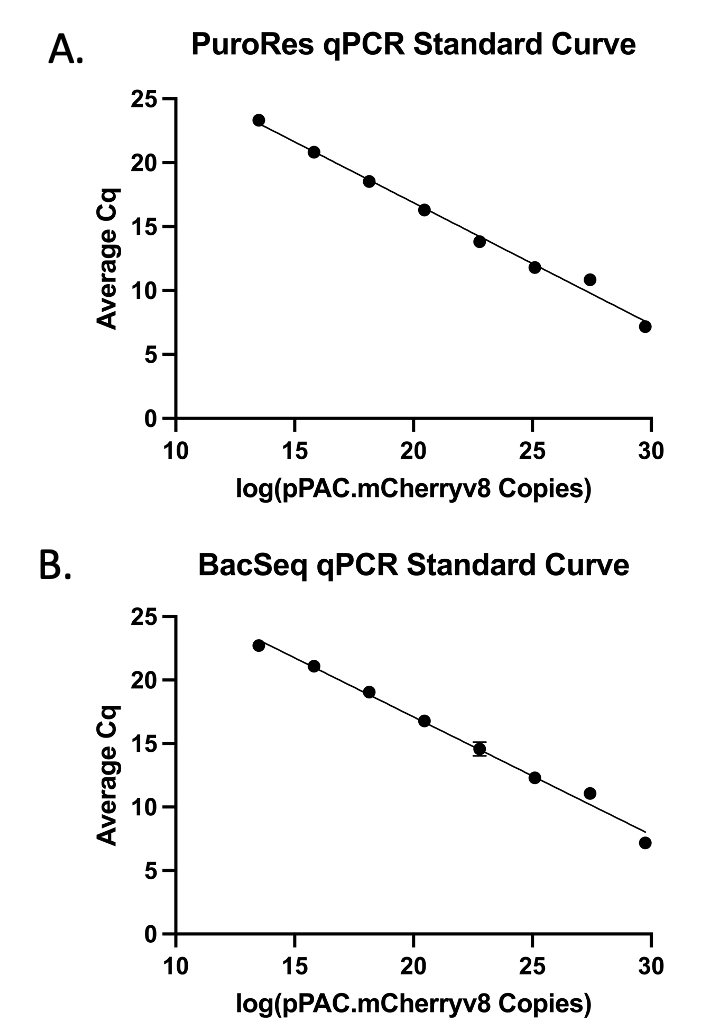

Supplement: Supplementary file 1 [file pathogens-15-00012-s001.zip › Supplmental Figure S2_.png]

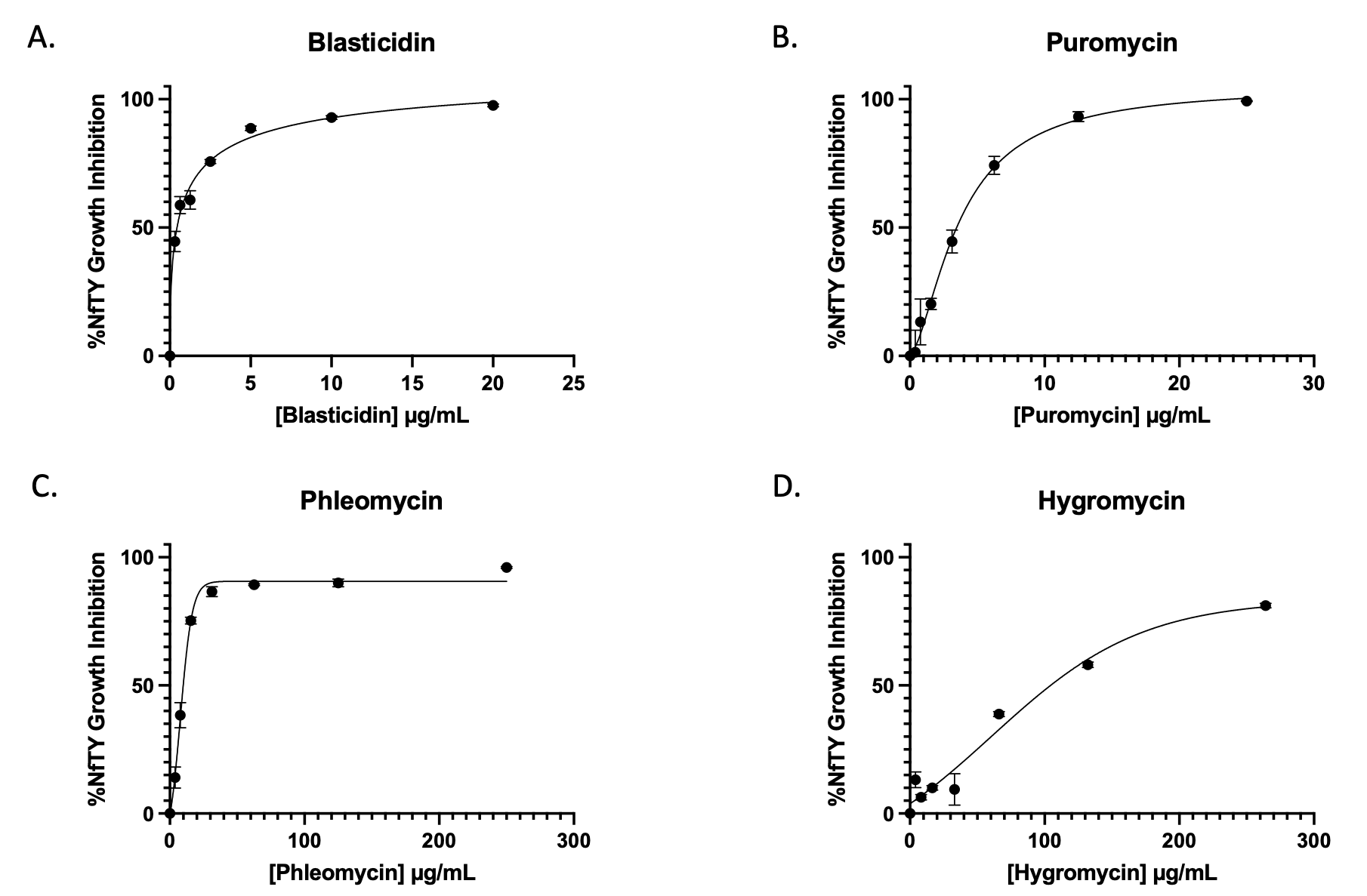

Supplement: Supplementary file 1 [file pathogens-15-00012-s001.zip › Supplmental Figure S3_.png]

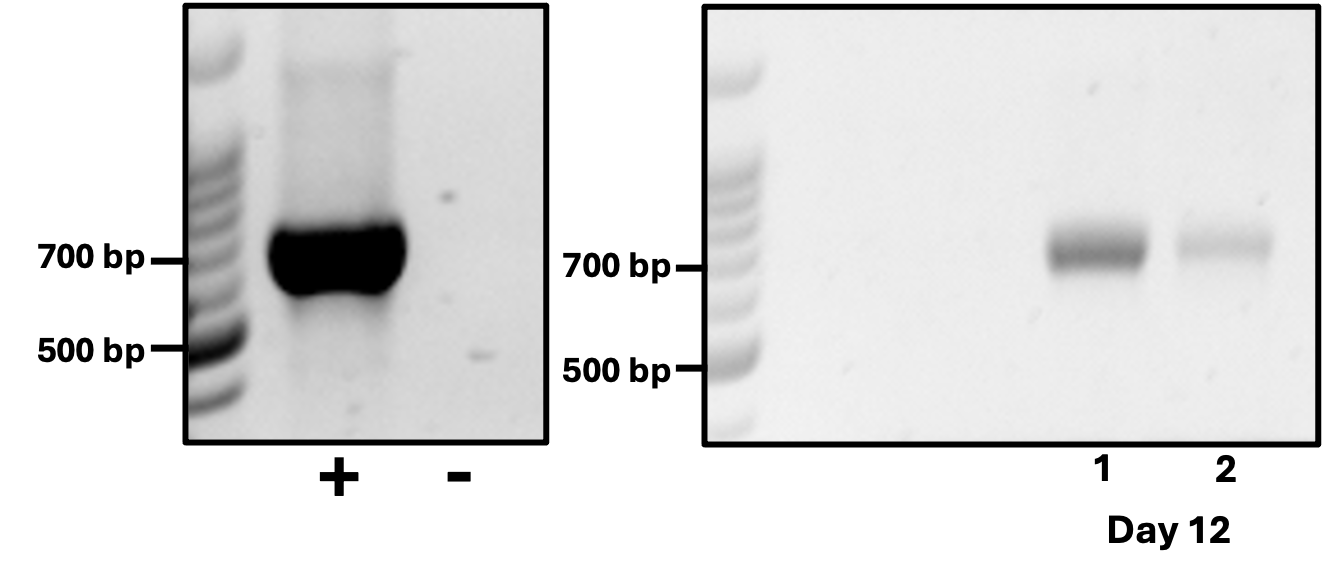

Supplement: Supplementary file 1 [file pathogens-15-00012-s001.zip › Supplmental Figure S4_.png]

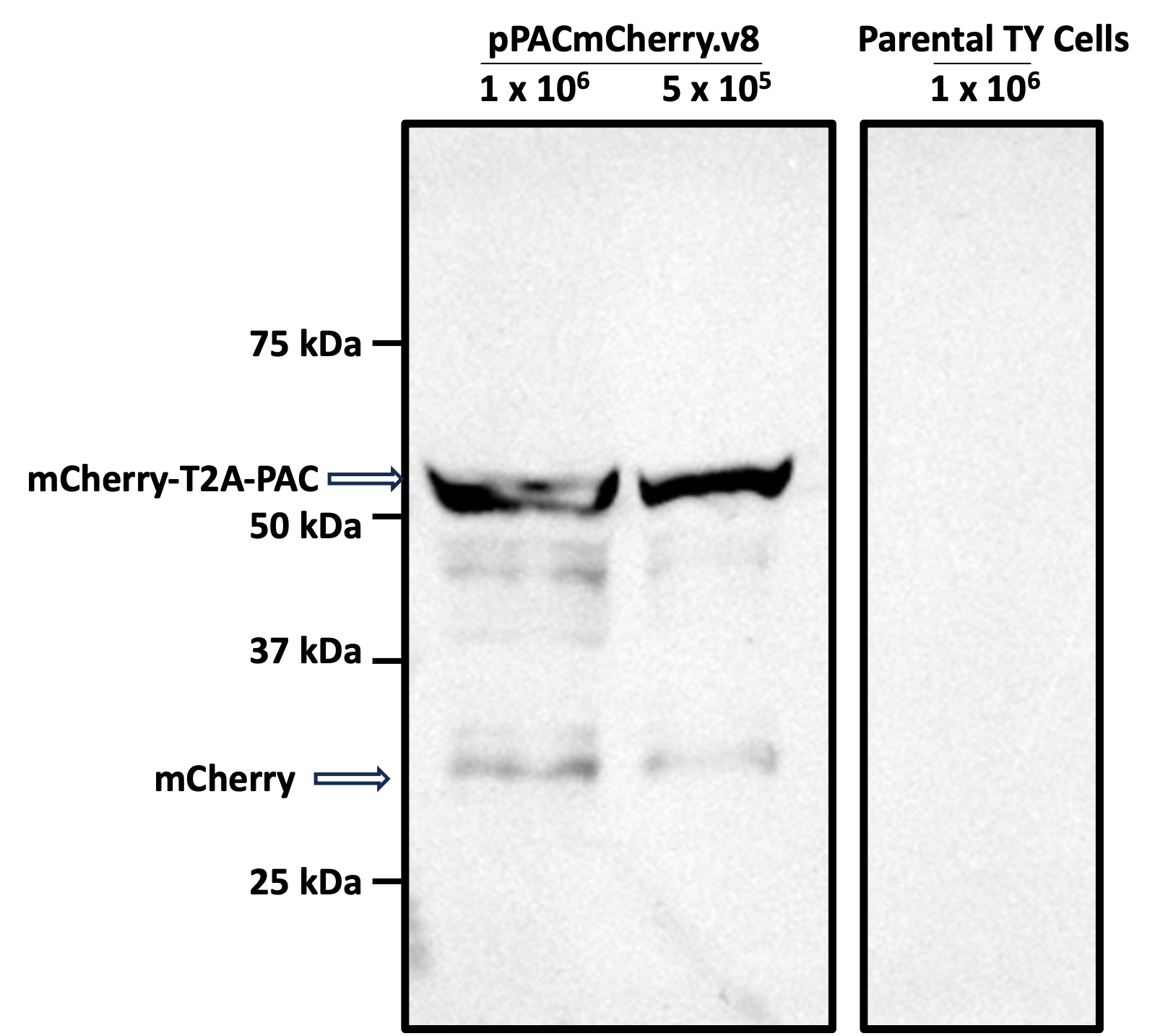

Supplement: Supplementary file 1 [file pathogens-15-00012-s001.zip › Supplmental Figure S5_.png]
